# Petroleum Hydrocarbon Bioremediation Using Native Fungal Isolates and Consortia

**DOI:** 10.1155/2021/6641533

**Published:** 2021-05-04

**Authors:** Asmaa A. Hamad, Hani A. Moubasher, Yasser M. Moustafa, Nermen H. Mohamed, Eman H. Abd-el rhim

**Affiliations:** ^1^Department of Botany and Microbiology, Faculty of Science, Cairo University, Cairo, Egypt; ^2^Department of Biology, Faculty of Science, Taif University, Taif, Saudi Arabia; ^3^Egyptian Petroleum Research Institute, Nasr City, Cairo, Egypt

## Abstract

Crude oil spills as a result of natural disasters or extraction and transportation operations are common nowadays. Oil spills have adverse effects on both aquatic and terrestrial ecosystems and pose a threat to human health. This study have been concerned with studying the capability of six fungal species (*Curvularia brachyspora*, *Penicillium chrysogenum*, *Scopulariopsis brevicaulis*, *Cladosporium sphaerospermum*, *Alternaria alternata*, and *Stemphylium botryosum*) and three fungal consortia (FC), FC1 (*P. chrysogenum* and *C. brachyspora*), FC2 (*S. brevicaulis* and *S. botryosum*), and FC3 (*S. brevicaulis*, *S. botryosum,* and *C. sphaerospermum*), to remediate petroleum hydrocarbons (PHs). Qualitative and quantitative changes in polyaromatic hydrocarbons (PAHs) and saturated hydrocarbons (SH) mixtures and the patterns of PHs degradation have been examined using HPLC and GC. Studying the GC chromatogram of *C. sphaerospermum* revealed severe degradation of SHs exhibited by this species, and the normal-paraffin and isoparaffin degradation percentage have been valued 97.19% and 98.88%, respectively. *A. alternata* has shown the highest significant (at *P* ˂ 0.05) PAH degradation percent reaching 72.07%; followed by *P. chrysogenum,* 59.51%. HPLC data have revealed that high-molecular-weight PAH percent/total PAHs decreased significantly from 98.94% in control samples to 68.78% in samples treated with *A. alternata*. FC1 and FC2 consortia have exhibited the highest significant PH deterioration abilities than did the individual isolates, indicating that these fungal consortia exhibited positive synergistic effects. The study supports the critical idea of the potential PAH and SH biodegradation as a more ecologically acceptable alternative to their chemical degradation.

## 1. Introduction

Environment contamination with petroleum hydrocarbons (PHs) has occurred since ancient time naturally, but in the latest years, man-made oil spills have become common [1]. PHs have been ordered as organic pollutants with mutagenic, carcinogenic, and toxic effects [2]. Even though physical and chemical processes, such as dilution, dispersion, sorption, abiotic transformations, and volatilization, are important means of crude oil removal, bioremediation treatment is the main promising mechanism for pollutants cleanup [3–5]. The commonly used chemical method for PHs remediation is the application of compounds such as cleaners, dispersants, biosurfactants, demulsifiers, and soil oxidizers, but due to their high cost and potential for causing secondary pollution as well as the toxicity of these chemicals to various ecosystems, there are widespread concerns about their applications [6]. Using natural microorganism populations in bioremediation processes is the utmost acceptable mechanism by which several xenobiotic contaminants, including PHs, have been removed from the environment [7]. Enhancement of indigenous microorganism growth, biostimulation, and external petrol-degrading microorganism inoculation are encouraging ways of hastening detoxifying and degrading activities in areas polluted with PHs with the lowest influence on the organization of the ecosystems [8, 9]. Crude oil contains varied chemical compounds including asphaltic compounds, heterocyclic, normal alkanes, cyclo- and isoalkanes, aromatics, and polycyclic aromatics. These compounds have a dissimilar degradation rate in the environment. The biodegradation potential was governed with the chemical structure of each compound, but further factors such as toxicity, volubility, and interaction with other molecules also affect the extent and the rate of biodegradation [10].

Effectiveness of bioremediation is based on microorganisms' capability to oxidize crude oil hydrocarbons enzymatically [11]. The grade of hydrocarbon degradation correlates with increase in microorganism populations and oxygenase activity [12]. Oxidative reactions are mediated by the enzymes catalase, dehydrogenase, urease, polyphenol peroxidase, and oxidase [13]. The microbial oxidation of crude oil hydrocarbons proceeds through a sequence of catalytic reactions yielding transitional metabolic products, alcohols, aldehydes, ketones, fatty, and carboxylic acids, which are eventually oxidized into CO_2_ [14]. Applying the ability of indigenous microorganisms for bioremediation and cleaning of contaminants is practicable and has cost-effective values [15, 16]. The prime objective of the current investigation was the evaluation of bioremediation effectiveness of the potent oil-degrading fungal strains, isolated from oil-polluted soils, in single and consortia cultures.

## 2. Materials and Methods

### 2.1. Sources of Soil Samples

**Figure 1:**
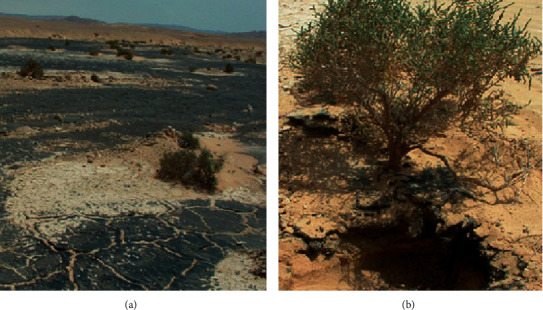
Soil polluted with petroleum oil as a result of accidental cracking of petrol pipelines. Soil samples, for isolation of potent petroleum-degrading fungi, have been assembled from open polluted areas (a) and from the rhizosphere of survived desert vegetation (b).

For isolation of the potent petrol-degrading fungi, the soil samples have been acquired from Asal oil field, Wadi Sidr (Al-Haitan), Ras-Sidr, West Sinai, Egypt. The soil has been polluted with PHs due to accidental cracking of petrol pipelines. Soil samples have been assembled randomly in presterilized glass bottles from different localities (rhizosphere of survived desert vegetation, a distance of 5–10 cm away from the plants, and from open polluted areas), 1–3 cm below the soil surface (Figure 1).

### 2.2. Isolation of Potential Oil-Degradation Fungi

The soil samples have been sorted carefully for removal of stones and further unwanted remains via a 2 mm sieve. For screening fungi of oil-degrading capacity, the method of serial dilution has been used. One gram soil has been sequentially diluted up to 10^−3^. Solidified Czapek´s Dox medium devoted of sucrose (constituents: potassium chloride, 1; sodium nitrate, 2; dipotassium hydrogen phosphate, 1; ferrous sulfate heptahydrate, 0.01; magnesium sulfate hepta-hydrate, 0.5; and agar, 20 g/l. The pH value was 5.5) has been used for screening the oil-degrading fungi. The medium has been poured in 9 cm diameter plates and covered with layer of 0.3 ml petroleum oil. Plates have been incubated for 7 days at 28°C after inoculation with three soil dilutions (10^−1^, 10^−2^, and 10^−3^), in triplicates. The observed criteria, after incubation, were the change in the appearance of oil or the production of visibly clear zone surrounding the fungi colonies. The potential oil-degrading isolates have been aseptically subcultured and tested again individually, for confirmation, on solidified Czapek´s Dox medium supplemented with PHs as the only carbon source.

### 2.3. Identification of Potent Fungal Isolates

Seven cultures of potential PH-degrading fungi (*Aspergillus flavus; Curvularia brachyspora*; *Penicillium chrysogenum*; *Scopulariopsis brevicaulis*; *Cladosporium sphaerospermum*; *Alternaria alternata*; and *Stemphylium botryosum*) were identified in the Assiut University Moubasher Mycological Center (AUMMC), regarding to the methods of Moubasher [17], using external features of the colony and microscopic examinations.

### 2.4. Quantitative Assay of PH Degradation

To get fungal spore suspension, six of the identified fungal species with oil-degradation capacity have been subcultured individually onto slants containing solidified Czapek´s Dox medium and incubated for 5 days in dark at 28°C. Spores have been suspended from each culture slant in 5 ml sterile distilled water. Spore suspensions have been diluted with double-sterile water to obtain similar cell counts of 1.0 × 10^4^ CFU/50 ml. One ml of individual spore suspension has been inoculated into 500 ml flasks containing 200 ml broth Czapek´s Dox medium free of sucrose and supplemented with 1 g petroleum oil as an alternative source of carbon. Inoculation of each fungus spore suspension has been carried out in triplicate prior to incubation on a shaking incubator for 30 days at 28°C. Flasks without any culture inoculation have been prepared, in three replicates, as a control.

### 2.5. Biodegradation Assay of Fungal Consortia

To obtain mixed cultures between previously selected six identified fungal species, they have been grouped into three consortia: the first consortia (designated FC1) (*Penicillium chrysogenum* and *Curvularia brachyspora*), second consortia (designated FC2) (*Scopulariopsis brevicaulis* and *Stemphylium botryosum*), and the third consortia (designated FC3) (*Scopulariopsis brevicaulis*, *stemphylium botryosum,* and *cladosporium sphaerospermum*).

*S. botryosum, S. brevicaulis, C. brachyspora, P. chrysogenum, C. sphaerospermum,* and *A. alternata* have been subcultured individually onto slants containing solidified Czapek´s Dox medium and incubated for 7 days in dark at 28°C. Spores have been suspended from each culture slant in 5 ml sterile distilled water, and then, spore suspensions for each group have been mixed. Spore suspensions have been diluted with double-sterile water to obtain similar cell counts of 1.0 × 10^4^ CFU/50 ml. One ml of each spore suspension mixture has been inoculated into 500 ml flasks contain 200 ml broth Czapek´s Dox medium free of sucrose and supplemented with 1 g petroleum oil as an alternative source of carbon. Inoculation of each fungal consortium has been performed in 3 replicates prior to incubation on a shaking incubator for 30 days at 28°C. Flasks containing 200 ml broth Czapek's Dox medium free of sucrose and supplemented with 1 g petroleum without any fungal culture inoculation have been prepared, in three replicates, as a control.

### 2.6. Extraction and Fractionation of Residual PHs

After incubation of fungal cultures, the PHs remaining has been extracted from liquid media using (1 : 1) dichloromethane. An equivalent quantity of dichloromethane has been mixed with the content of each flask. The PH content of each flask has been extracted using a separating funnel, by gentle shaking. The lower organic layer has been gathered (after filtering to get rid of fungal growths) into a dry clean conical flask. Each culture has been extracted three times, and then, extracts have been collected and evaporated completely using a vacuum pump, in a 40°C water bath. The percent of PHs lost using this method was found to be 4.57% using positive control.

The PH components have been fractionated using the column chromatography method [18]. 50 gram activated silica gel (mesh 60–120) has been loaded in a glass column (1.5 cm in diameter and 100 cm height). The extracted PHs have been dissolved in n-hexane and divided into insoluble and soluble portions. The insoluble asphaltene fraction has been separated by n-hexane deposition, while the soluble fraction has been loaded on the top of the silica gel column and then eluted with solvents of various polarities. 200 ml n-hexane has been used to elute the saturated hydrocarbon (SHs) fraction, followed by 150 ml benzene for obtaining aromatic fraction. It is followed by application of a 100 ml (1 : 1) benzene and methanol mixture to elute resin (polar) fraction.

### 2.7. Saturated Hydrocarbons Analysis

The change in the mixture of SH fractions has been estimated by Perkin Elemer (Clarus 500) gas chromatography (GC). The apparatus has been supplied with a fused silica capillary column (60 m length × 0.32 mm i.d), loaded with poly (dimethyl siloxane) HP-1 (nonpolar packing) of 0.5 *μ*m film thickness and a hydrogen flame ionization detector. The column temperature has been programmed from 100 to 300°C at a constant rate of 3°C/min, and nitrogen (oxygen-free) has been used as a carrier gas with a flow rate of 2 ml/min. The chromatograph injector has been heated at 350°C, while the detector has been heated at 350°C and operated with an adjusted hydrogen flow rate to optimize the detector acuteness. The sample has been melted, and 0.1 *μ*l of it has been subjected into the injector. A pure normal-paraffin (n-paraffin) mixture has been used as standard. The peak area of each resolved part (containing either n- and isoparaffin) has been individually evaluated, while the hump consisting mainly of cycloparaffins and aromatics with long side chains has been estimated as total values.

### 2.8. Polyaromatic Hydrocarbons (PAHs) Analysis

Qualitative and quantitative changes in PAH mixtures have been achieved using high-performance liquid chromatography (Waters HPLC 600E) for aromatic fractions. PAH standards purchased from Supelco CRMs for chromatography solutions (https://www.sigmaaldrich.com/analytical-chromatography/analytical-products.html?TablePage=120241095) have been also subjected successively to be analyzed by high-performance liquid chromatography (HPLC). The apparatus consists of an autosampler, Waters 717 plus, attached to a computerized system with Millennium 3.2 software and a dual UV absorbance detector, Waters 2487. The separation has been performed according to the conditions mentioned in [19].

### 2.9. Calculations and Statistical Analysis

- % degradation of total SHs in sample_(i)_ = ((total SHs in the control sample−total SHs in sample_(i)_)/total SHs in the control sample) *∗* 100
- % degradation of n-paraffins in sample_(i)_ = ((total n-paraffins in the control sample−total n-paraffins in sample_(i)_)/total n-paraffins in the control sample) *∗* 100
- % degradation of isoparaffins in sample_(i)_ = ((total isoparaffins in thee control sample−total isoparaffins in sample_(i)_)/total isoparaffins in the control sample) *∗* 100
- % degradation of total PAHs in sample_(i)_ = ((total PAHs in the control sample − total PAHs in sample_(i)_)/total PAHs in the control sample) *∗* 100
- % of high-molecular-weight PAHs (% HMWHs) of sample_(i)_ = (total HMWHs in sample_(i)_/total PAHs in sample_(i)_) *∗*100
- % of low-molecular-weight PAHs (% LMWHs) of sample_(i)_ = (total LMWHs in sample_(i)_/total PAHs in sample_(i)_) *∗* 100

Analysis of variance (ANOVA) has been achieved using SPSS-20 statistical software package for PCs. Multiple comparisons of means for experimental parameters have been estimated (at a reliability level of *P* < 0.05) using Duncan's multiple range test. All experiments have been conducted in triplicates and are represented as mean ± standard error (SE).

## 3. Results

### 3.1. Identification of Fungi with Oil-Degradation Ability

**Figure 2:**
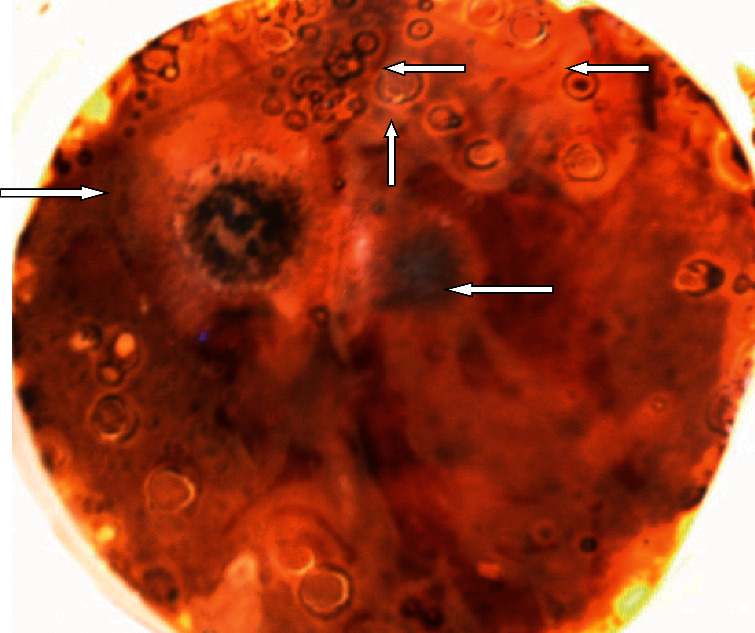
Microbial colonies that were screened on solidified Czapek's Dox medium complemented with petroleum hydrocarbons as a solitary carbon source have shown a clear modification in the appearance of petroleum oil surrounding them.

**Figure 3:**
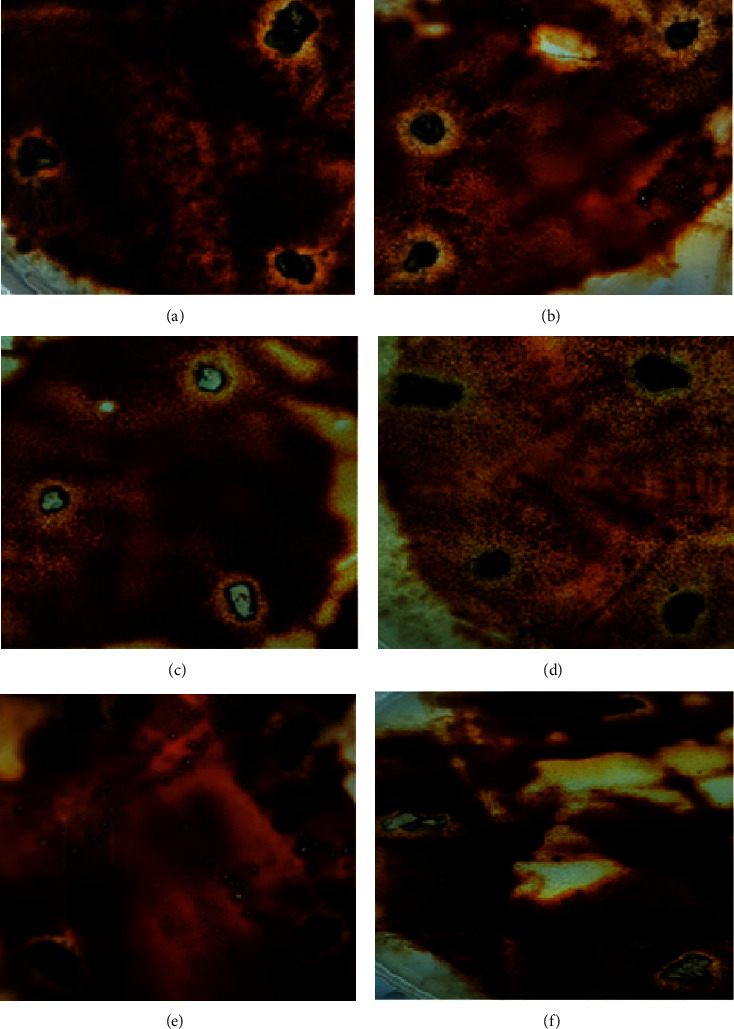
Fungal isolates that were grown on solidified Czapek's Dox medium complemented with petroleum hydrocarbons as a solitary carbon source have shown a clear modification in the appearance of petroleum oil surrounding them. (a) *Penicillium chrysogenum*; (b) *Scopulariopsis brevicaulis*; (c) *Stemphylium botryosum*; (d) *Alternaria alternate*; (e) *Cladosporium sphaerospermum;* and (f) *Curvularia brachyspora.*

Screening of fungi on solidified Czapek´s Dox medium supplemented with PHs as a sole carbon source showed quite a few fungi colonies that have been able to change the appearance of the petrol surrounding them (Figure 2). Fungal isolates that showed the ability to change the appearance of petrol, or producing of a visible clear zone surrounding their colonies, have been tested individually, for confirmation, on the same previously mentioned medium (Figures 3(a)–3(f)).

Seven fungal isolates that showed capabilities for PH biodegradation have been identified as follows: *Aspergillus flavus* Link (AUMMC 11554); *Curvularia brachyspora* Boedijn (AUMMC 11555); *Penicillium chrysogenum* Thom (AUMMC 11556); *Scopulariopsis brevicaulis* (Saccardo) Bainier (AUMMC 11560); *Cladosporium sphaerospermum* Penzig (AUMMC 11561); *Alternaria alternata* (Fries) Keissler (AUMMC 11562); and *Stemphylium botryosum* Wallroth (AUMMC 11564).

### 3.2. Changes in SH Mixture upon Using the Individual Fungal Strains

**Figure 4:**
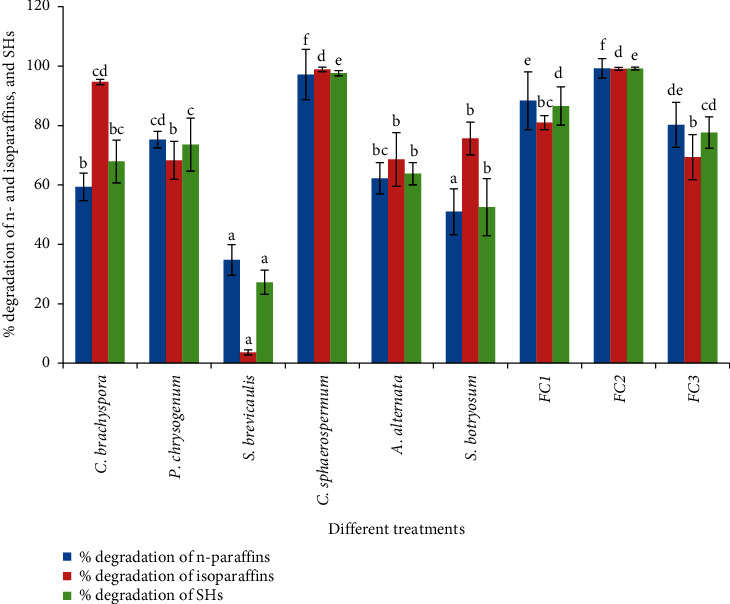
% degradation of n-paraffins, isoparaffins, and total SHs, estimated by GC, upon using pure and mixed fungal cultures corresponding to untreated control. SE is presented as a vertical bar above each mean. The same letters indicate that there is no significant difference in % degradation (of n-paraffin, isoparaffin, and SHs) in samples treated with different pure and mixed fungal cultures regarding Duncan's multiple range tests at *P* < 0.05. FC1 **=** mixed culture of *Penicillium chrysogenum* and *Curvularia brachyspora*, FC2 **=** mixed culture of *Scopulariopsis brevicaulis* and *Stemphylium botryosum*, and FC3 **=** mixed culture of *Scopulariopsis brevicaulis*, *Stemphylium botryosum,* and *Cladosporium sphaerospermum*.

The changes of SH fractions in the sample treated with pure fungal strains have been analyzed using gas chromatography (Table 1 & Figure 4). GC chromatograms for the SHs fractions presented that control samples (petroleum samples untreated with fungal cultures) exhibited a broad spectrum of regularly spaced normal-paraffin (n-paraffins) hydrocarbon peaks. Normal-Carbon14 (n-C14) has been found to be the initial carbon number in control samples, while in the samples treated with fungal cultures, for 30 days, n-C17 has been found to be the initial carbon number, except *S. botryosum* and *S. brevicaulis* (found to be n-C16), indicating that all the used fungal strains have succeeded in completely removing n-C14 and n-C15.

As shown in Figure 4, the filamentous fungus *C. sphaerospermum* has presented the highest significant percent of SH degradation, and the percent of n-paraffins and isoparaffins degradation valued 97.19% and 98.88%, respectively. Studying the characteristic features for *C. sphaerospermum* GC chromatogram has revealed that n-paraffin peaks have been either missed or presented as minor peaks representing severe degradation exhibited by the species after incubation with petroleum oil as a carbon source. Isoparaffin and n-paraffin degradation percent exhibited by *A. alternata* was almost the same, reaching 68.59% and 62.26%, respectively. Statistically, there has been no significant variance (at *P* ˂ 0.05) in SH degradation percent upon using *P. chrysogenum* in correspondence to *A. alternate*. The percent SH degradation has been valued 73.57% and 63.80% for *P. chrysogenum* and *A. alternata,* respectively.

GC chromatograms for the samples treated with *C. brachyspora* and *S. botryosum* have indicated considerable degradation percent of both isoparaffins and n-paraffins. The percent of n-paraffins degradation reached 59.38% and 51.01%, while the percent of isoparaffin degradation reached 94.67% and 75.64% by using *C. brachyspora* and *S. botryosum,* respectively. *S. brevicaulis* has shown the lowest significant percent degradation of isoparaffins and n-paraffins, amounting 3.67% and 34.75%, respectively.

### 3.3. Changes in the PAH Mixture upon Using the Individual Fungal Strains

**Figure 5:**
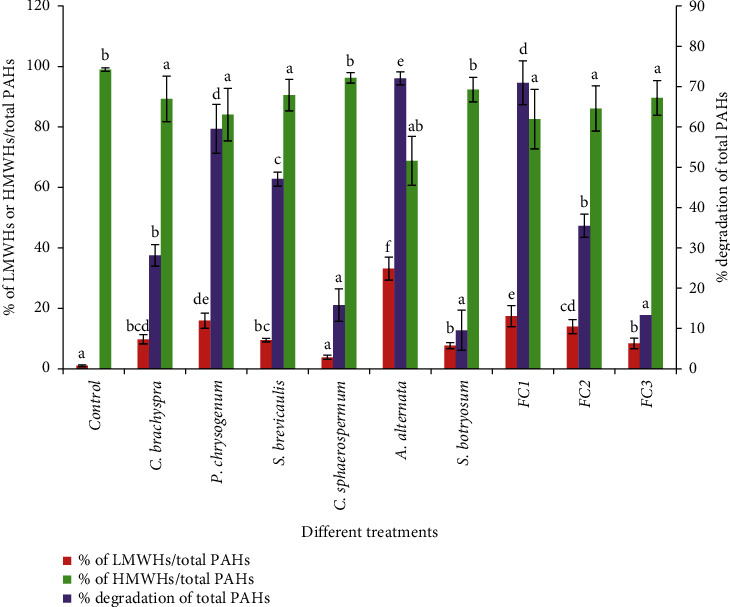
Quantitative degradation estimated by HPLC, of % LMWHs/total PAHs, % HMWHs/total PAHs, and % degradation of total PAHs using pure and mixed fungal cultures. SE is presented as a vertical bar above each mean. The same letters indicate that there is no significant difference in % of LMWHs and HMWHs/total PAHs and % degradation of PAHs in samples treated with different pure and mixed fungal cultures regarding Duncan's multiple range tests at *P* < 0.05. FC1 **=** mixed culture of *Penicillium chrysogenum* and *Curvularia brachyspora*, FC2 **=** mixed culture of *Scopulariopsis brevicaulis* and *Stemphylium botryosum*, and FC3 **=** mixed culture of *Scopulariopsis brevicaulis*, *Stemphylium botryosum,* and *Cladosporium sphaerospermum*.

Changes in the PAH mixture of the aromatic fractions are presented in Table 2 and Figure 5. PAHs have been divided according to ring number into high (consists of hexa-, penta-, and tetra-aromatic rings) and low (comprises of three and two aromatic rings) molecular-weight PAHs [20]. Estimation of PAHs for the control sample has shown the occurrence of both high- and low-molecular-weight PAHs. High-molecular-weight PAHs (HMWHs) have been predominant and exist in control samples in very high percentage, 44.83%, 38.65%, and 15.46% for tetra-, penta- and hexa-aromatic ring PAHs, respectively, whereas trace amounts of low-molecular-weight PAHs (LMWHs) have been reported.

Statistically, *A. alternata* has shown the highest significant total PAH degradation percent, reaching 72.07%, followed by *P. chrysogenum,* 59.51%. HPLC data revealed that the percent of HMWHs decreased from 98.94% in control samples to 68.78% in samples treated with *A. alternata*. On the other hand, the percent of LMWHs increased from 1.04% to 33.11%. Samples treated with *S. brevicaulis* and *C. brachyspora* have shown considerable percent of total PAHs degradation, reaching 47.07% and 28.14%, respectively. *C. sphaerospermum* also has shown detectable degradation percent of total PAHs (15.78%), and the percent of HMWHs decreased in correspondence to untreated control from 98.94% to 96.22%. Samples treated with *S. botryosum* have shown the lowest significant degradation percent of total PAHs, reaching 9.55%.

### 3.4. Changes in the SH and PAH Mixture upon Using Fungal Consortia

The changes in the mixture of SHs and PAHs upon using fungal consortia are illustrated in Tables 3 and 4 and Figures 4 and 5. The rate of SH and PAH degradation exhibited by fungal consortium FC1 has been significantly higher than that observed with the single cultures of *P. chrysogenum* and *C. brachyspora*. After 30 days of incubation, the percent degradation of SHs and PAHs exhibited upon using FC1 reached 86.57% and 70.93%, respectively, corresponding to 67.91% and 28.14% and 73.57% and 59.51% demonstrated with single cultures of *C. brachyspora* and *P. chrysogenum,* respectively.

The rate of SH degradation using a mixed culture of *S. brevicaulis* and *S. botryosum* (FC2) has been significantly higher than that observed with the single cultures and also higher than that demonstrated with fungal consortia FC1 and FC3. Percent degradation of SHs exhibited by FC2 impressively improved, reaching 99.18% corresponding to 27.24%, 52.53%, 86.57%, and 77.62% that demonstrated with *S. brevicaulis*, *S. botryosum*, FC1, and FC3, respectively. Conversely, there has been no detectable improvement for PAH degradation in samples inoculated with FC2 compared to that inoculated with single cultures of *S. brevicaulis* and *S. botryosum*.

The presence of *C. sphaerospermum* in combination with *S. brevicaulis* and *S. botryosum* (FC3) significantly decreased the efficiency of SH and PAH degradation from 97.60% to 47.07% using single cultures of *C. sphaerospermum* to 77.62% and 13.32%, respectively, using fungal consortium FC3.

## 4. Discussion

Crude petroleum oil is a complex mixture of hydrocarbons; within this mixture, hundreds of individual components can be recognized. Based on the relevant structures, many chemical categories are classified [21]. Many microbial species are distinguished by their ability to analyze PHs, and several authors demonstrated that a large variety of fungi and bacteria have this ability [18, 22, 23]. In the present study, the fungi isolated from PH-contaminated soil have shown significant capability for SH and PAH degradation. Our finding is in agreement with the work of Emmanuel et al. [24]; they reported that microorganisms isolated from oil-contaminated sites exhibited the highest activity for PH degradation than microorganisms isolated from uncontaminated sites. Lianos and Kioller [25] observed the effect of oil waste application on fungal populations. They found that oil application favored growth of oil-degrading species.

The important role of soil fungi in hydrocarbon oxidizing activities, consequently in the oil degradation activity, has been reported by many authors [3, 26]. However, the present study is a pioneer in estimating the capability of *Curvularia brachyspora*, *Penicillium chrysogenum*, *Scopulariopsis brevicaulis*, *Cladosporium sphaerospermum*, *Alternaria alternata*, and *Stemphylium botryosum* in biodegradation of highly toxic and carcinogenic benzo [a] anthracene, benzo [a] pyrene, benzo [b] fluoranthene, benzo [k] fluoranthene, chrysene, dibenzo (a,h) anthracene, and indeno (1,2,3-cd) pyrene using HPLC estimation. Benzo [a] pyrene has been classified as the first discovered carcinogenic compound [27].

According to the results obtained in the current study, the most potent strains in deterioration of SHs were the filamentous fungi *C. sphaerospermum* followed by *P. chrysogenum*. For PAH deterioration, *A. alternata* and *P. chrysogenum* were the most potent strains. The higher hydrocarbon degradation rates presented by these fungal species could be due to their fast enzymatic production response and the massive growth exhibited by the four strains during their growth phases [28]. Utilization patterns of hydrocarbons have been found to be similar for most tested fungal species; however, individual isolates have a considerable variability. The abilities of fungi and bacteria to degrade hydrocarbons have been studied by Walker et al. [29]; they found that yeasts and bacteria showed reducing efficiency to remediate long-chain alkanes, while filamentous fungi exhibited no favored remediation for chain lengths.

Steliga [30] reported that genus *Cladosporium* and genus *Penicillium* are among the most potent filamentous fungi participating in aliphatic hydrocarbon biodegradation, whereas fungi belonging to *Cunninghamella, Fusarium, Penicillinum*, and *Aspergillus* can take part in aromatic hydrocarbon decomposition. Obire et al. [31] reported strains of genus *Cladosporium* and *Penicillium* as significant soil fungi groups capable of utilizing PHs. Madani [32] illustrated that genus *Penicillium* and *Alternaria* are rich in crude-oil-assimilating strains. The degradation of PHs has also been studied by Ameen et al. [33]. The authors used GC-analysis for investigation of petroleum oil degradation using filamentous fungi, and they found that *C. sphaerospermum* strongly degraded tritetracontane, 1,2-benzenedicarboxylicacid,mono(2-ethylhexyl) ester, and tetrapentacontane, 1,54-dibromo.

*P. chrysogenum* has been previously recorded as a potent oil-utilizing species [34, 35]. Balaji et al. [36] studied the GC profile for soil contaminated with PHs after 28 days of incubation with *P. chrysogenum,* and the authors have reported complete n-alkane degradation. Isoprenoids such as pristine (C17) and pyrene (C18) have shown a considerable degradation rate. Verma et al. [37] reported the capability of *A. alternata* to degrade the PAHs compounds naphthalene, phenanthrene, and anthracene. The authors proposed that *A. alternata* could degrade the hydrocarbon by producing manganese-dependent peroxide and polyphenol oxidase enzyme.

This is the first study to describe the potentiality of FC1 (*P. chrysogenum* and *C. brachyspora*), FC2 (*S. brevicaulis* and *S. botryosum*), and FC3 (*S. brevicaulis*, *S. botryosum,* and *C. sphaerospermum*) consortia for PH degradation using GC and HPLC analysis. Fungal consortia FC1 and FC2 have exhibited more oil degradation ability than did the individual isolates. The results have shown that FC1 and FC2 consortia demonstrated positive synergistic effects. A wide range of metabolic and physiological factors are essential for degradation of different hydrocarbons. Usage of fungal consortia could result in metabolic integration as the metabolites created by a specific fungus through incomplete degradation may be used as a substrate by another fungus, increasing the possibility of hydrocarbons remediation effectively [38]. Adebusoye et al. [39] illustrated that mixed cultures are required for effective biodegradation of crude petroleum oil because the hydrocarbon mixtures vary markedly in solubility, volatility, and susceptibility to degradation.

The lowest crude oil degradation exhibited by using FC3 could be due to the fungal growth reduction as a result of many factors such as the competition and antagonisms [40]. These results are in accordance with the finding obtained by AI-Dossari [41]; the author founded that mixed fungal isolates (*Aspergillus niger, Aspergillus fumigatus, Peniclllium funiculosum,* and *Fusarium solani*) exhibited reductions in PH biodegradation despite the high rate of hydrocarbon degradation by each single isolate separately.

The data in the present study have shown a significant SH degradation percent, exhibited by all isolates, particularly *C. sphaerospermum, P. chrysogenum, C. brachyspora,* and *A. alternata*. The SH fraction consists of normal, branched, and cycloalkanes (naphthenes). The route of n-alkane biodegradation through a monoterminal attack with alcohol formation followed by the creation of aldehyde and a monocarboxylic acid has been demonstrated by Nkwelang et al. [42]. Jurtehuk and Cardini [43] reported that Omega (diterminal) oxidation also occurred.

N-paraffin and isoparaffin degradation percent exhibited by *C. sphaerospermum, P. chrysogenum,* and *A. alternata* in the present was study almost the same, indicating that both n-paraffin and isoparaffin have been attacked in the same rate by these strains. Pirnik and McKenna [44] reported highly branched alkanes, isoprenoid, subjected to omega oxidation as the major pathway of degradation; however, methyl branching at the position of beta blocks f-oxidation and additional strategy may be required such as omega oxidation, alpha oxidation, or removal of the beta alkyl group.

Cycloalkanes are more resilient to attack by microorganisms; however, there were several reports considering the fungal metabolism of cycloalkanes and correlated compounds [45]. Cobet and Guard [46] illustrated that up to six-membered condensed-ring cycloalkanes have been subjected to degradation by microorganisms. Several unsubstituted cycloalkanes have been described to be attacked by microbial co-oxidation with alcohol or ketone formation, followed by ring cleavage [47].

Several authors reviewed the microbial degradation of aromatic hydrocarbons [18]. Aromatic compound degradation by microbial activities usually involves formation of a trans-diol, ring cleavage, and then, formation of a diacid [48]. Cripps and Watkinson [49] reported that extensive substitution of methyl branching may inhibit initial oxidation. Condensed aromatic ring structures have been reported to be attacked by microbial enzymes by a similar monocyclic structure metabolic pathway [50]. Walker et al. [51] described that condensed structures with four or more rings may be attacked by co-oxidation in some cases.

## 5. Conclusions

This study has succeeded in isolating a large number of petroleum-hydrocarbon-degrading taxa. Based on the results of the laboratory-scale studies, *C. sphaerospermum* and *P. chrysogenum* presented the highest SH degradation percentage while *A. alternata* and *P. chrysogenum* exhibited the highest degradation efficiency of PAHs. It is recommended to use mixed cultures of *P. chrysogenum* and *C. brachyspora* for enhanced degradation of both SHs and PAHs. This study supports the critical idea of the potential PAH biodegradation as more a ecologically acceptable alternative to their chemical degradation. After large-scale production, the designated single organisms or the fungal consortium FC1 can be used, in field application, for PHs remediation.

## Figures and Tables

**Table 1:** Quantitative degradation analysis, estimated by GC, of saturated hydrocarbons using six fungal species corresponding to untreated control.

**Carbon Number**	**Control**	***C. brachyspora***	***P. chrysogenum***	***S. brevicaulis***	***C. sphaerospermum***	***A. alternata***	***S. botryosum***

**C14**	1396.35	0.00	0.00	0.00	0.00	0.00	0.000
**C15**	5506.49	0.00	0.00	0.00	0.00	0.00	0.000
**C16**	9741.55	0.00	0.00	354.91	0.00	0.00	420.80
**C17**	1.77/10000	2052.19	130.72	5088.91	52.35	320.23	2769.74
**C18**	1.80/10000	4332.79	628.76	4396.94	428.01	2104.39	5292.99
**C19**	1.13/10000	2918.78	680.54	3180.60	337.42	1621.83	5170.32
**C20**	1.10/10000	3375.22	1212.27	3501.80	380.96	1822.72	4202.27
**C21**	9653.44	3787.89	1474.04	4310.44	381.22	1828.92	2780.02
**C22**	8929.10	2780.84	1772.12	4865.36	311.14	2086.02	2972.01
**C23**	7804.85	2064.85	1460.82	3002.51	142.30	2071.79	3089.31
**C24**	7228.75	1674.47	1678.62	3133.56	97.53	2302.22	2077.54
**C25**	5704.98	69.11	1426.26	3108.41	55.98	1824.42	1937.97
**C26**	4752.65	758.97	1261.27	2370.72	34.45	1736.39	1302.14
**C27**	3005.48	0.000	1055.78	2050.27	21.02	1738.55	856.60
**C28**	3301.36	1856.57	1197.27	2267.14	13.79	1965.49	1678.97
**C29**	3688.51	1997.92	1194.33	2833.07	8.84	1868.91	1363.89
**C30**	1777.19	1324.52	1211.70	2128.80	10.88	1488.06	767.17
**C31**	2424.01	1701.75	895.48	1606.24	9.36	768.38	361.18
**C32**	815.56	873.42	850.12	1110.72	3.93	692.67	294.89
**C33**	2007.55	1062.6	769.47	1067.63	3.45	901.76	915.34
**C34**	1670.29	164.94	276.43	1187.96	2.8	1135.26	534.65
**C35**	1226.69	0.00	611.64	1324.32	0.00	1166.39	585.71
**C36**	337.78	0.00	208.18	359.38	0.00	814.50	0.00
**C37**	222.98	0.00	188.43	0.00	0.00	270.54	0.00
**C38**	416.02	0.00	0.00	0.00	0.00	263.20	0.00
Total n-parafin	**81611.66**	**33150.53**	**20184.19**	**53249.70**	**2295.42**	**30792.61**	**39979.52**
% degradation of n-parafin		**59.38**	**75.27**	**34.75**	**97.19**	**62.26**	**51.01**
Total isoparafin	**26009.72**	**1384.3**	**8255.48**	**25055.53**	**290.82**	**8168.402**	**6337.16**
%degradation of isoparafin		**94.67**	**68.26**	**3.67**	**98.88**	**68.59**	**75.64**
Total saturated hydrocarbon	**107621.38**	**34534.83**	**28439.67**	**78305.23**	**2586.24**	**38961.01**	**45710.68**
**%**degradation of saturated hydrocarbon		**67.91**	**73.57**	**27.24**	**97.60**	**63.80**	**52.53**

**Table 2:** Changes in the mixture of PAHs (mg/l), estimated by HPLC, for samples treated with six fungal species corresponding to untreated control. Nap = naphthalene, *A* = acenaphthylene, Ace = acenaphthene, F = fluorene, Phe = phenanthrene, Ant = anthracene, Flu = fluoranthene, Pyr = pyrene, BaA = benzo (a) anthracene, BaP = benzo (a) pyrene, BbF = benzo (b) fluoranthene, BkF = benzo (k) fluoranthene, Chr = chrysene, DahA = dibenzo (a,h) anthracene, and BP = benzo (g, h, i) perylene.

Number of rings	PAHs	Control	***C.***	***P.***	***S.***	***C.***	***A.***	***S.***
			b***rachyspora***	***chrysogenum***	***brevicaulis***	***sphaerospermum***	***alternata***	***botryosum***
2	Naph.	0.34	0.30	0.59	0.20	0.55	0.00	0.00
% of 2 rings/total PAHs		0.12	0.15	0.51	0.13	0.23	0.51	0.00
3	A.	0.08	0.30	0.00	0.04	0.00	11.51	3.10
	Ace.	0.00	14.00	6.99	6.51	0.00	0.00	1.66
	F.	2.09	0.00	0.00	0.00	2.58	0.00	3.36
	Phe.	0.00	3.63	5.54	3.34	2.87	9.06	8.56
	Ant.	0.44	1.63	5.13	4.18	3.01	5.64	2.91
% of 3 rings/total PAHs		0.92	9.61	15.40	9.38	3.55	33.11	7.65
4	Flu.	30.41	46.62	7.64	15.12	52.62	14.46	36.45
	Pyr.	50.30	3.28	20.92	24.95	38.62	0.00	7.98
	BaA.	4.70	47.20	10.49	15.27	5.04	5.38	94.56
	Chr.	41.61	15.05	15.30	20.80	2.54	3.31	13.60
% of 4 rings/total PAHs		44.83	55.07	47.36	50.77	41.40	29.24	59.54
5	BbF.	26.11	26.25	22.51	6.59	42.06	13.48	11.52
	BkF.	19.74	19.39	13.27	7.51	37.52	16.08	27.89
	BaP.	1.99	9.87	0.08	0.00	11.04	1.74	7.57
	DahA.	61.68	4.13	0.00	39.50	3.70	0.00	37.12
% of 5 rings/total PAHs		38.65	29.28	31.25	35.74	39.52	39.54	32.81
6	Bp.	24.57	4.70	0.00	0.95	20.00	0.00	0.00
	Indeno	19.24	5.30	6.28	5.03	16.50	0.00	0.00
% of 6 rings/total PAHs		15.46	4.91	5.47	3.98	15.30	0.00	0.00
	Total PAHs	283.36	203.64	114.73	149.98	238.65	79.16	256.29
% degradation of total PAHs			28.14	59.51	47.07	15.78	72.07	9.55

**Table 3:** Quantitative degradation analysis, estimated by GC, of saturated hydrocarbons using three fungal consortia corresponding to untreated control. FC1 **=** mixed culture of *Penicillium chrysogenum* and *Curvularia brachyspora*, FC2 **=** mixed culture of *Scopulariopsis brevicaulis* and *Stemphylium botryosum*, and FC3 **=** mixed culture of *Scopulariopsis brevicaulis*, *Stemphylium botryosum,* and *Cladosporium sphaerospermum*.

Carbon number	Control	FC1	FC2	FC3
**C14**	1396.35	0.00	0.00	0.00
**C15**	5506.49	0.00	0.00	0.00
**C16**	9741.55	0.00	0.00	0.00
**C17**	1.77/10000	983.38	0.00	1430.33
**C18**	1.80/10000	1409.57	0.00	1657.65
**C19**	1.13/10000	879.87	0.00	656.45
**C20**	1.10/10000	769.83	10.59	656.65
**C21**	9653.44	473.86	4.87	967.54
**C22**	8929.10	453.47	67.27	867.64
**C23**	7804.85	786.65	81.78	767.57
**C24**	7228.75	215.85	88.56	404.73
**C25**	5704.98	743.87	13.68	748.58
**C26**	4752.65	637.84	36.47	574.56
**C27**	3005.48	675.50	27.45	537.48
**C28**	3301.36	32.31	87.98	865.65
**C29**	3688.51	453.52	93.67	532.75
**C30**	1777.19	89.75	12.73	864.47
**C31**	2424.01	108.65	19.38	759.42
**C32**	815.56	277.76	15.74	834.46
**C33**	2007.55	234.98	0.00	776.54
**C34**	1670.29	76.41	0.00	497.58
**C35**	1226.69	45.36	45.00	623.78
**C36**	337.78	156.47	38.00	498.18
**C37**	222.98	0.00	0.00	265.67
**C38**	416.02	0.00	0.00	332.43
Total n-parafin	**81611.66**	**9504.9**	**643.17**	**16120.11**
% degradation of n-parafin		**88.35**	**99.21**	**80.25**
Total isoparafin	**26009.72**	**4949.67**	**243.65**	**7964.87**
%degradation of isoparafin		**80.97**	**99.06**	**69.38**
Total saturated hydrocarbon	**107621.38**	**14454.57**	**886.82**	**24084.98**
%degradation of saturated hydrocarbon		**86.57**	**99.18**	**77.62**

**Table 4:** Changes in the mixture of PAHs (mg/l), estimated by HPLC, for samples treated with three fungal consortia corresponding to untreated control. FC1 **=** mixed culture of *Penicillium chrysogenum* and *Curvularia brachyspora*, FC2 **=** mixed culture of *Scopulariopsis brevicaulis* and *Stemphylium botryosum*, and FC3 **=** mixed culture of *Scopulariopsis brevicaulis*, *Stemphylium botryosum,* and *Cladosporium sphaerospermum*. Nap = naphthalene, *A* = acenaphthylene, Ace = acenaphthene, *F*= fluorene, Phe = phenanthrene, Ant = anthracene, Flu = fluoranthene, Pyr = pyrene, BaA = benzo (a) anthracene, BaP = benzo (a) pyrene, BbF = benzo (b) fluoranthene, BkF = benzo (k) fluoranthene, Chr = chrysene, DahA = dibenzo (a, h) anthracene, and BP = benzo (g, h, i) perylene.

Number of rings	PAHs	Control	FC1	FC2	FC3
2	Naph.	0.34	0.00	0.00	0.00
% of 2 rings/total PAHs		0.121	0.00	0.00	0.00
3	A.	0.08	0.00	0.02	0.21
	Ace.	0.00	9.87	24.45	19.24
	F.	2.09	1.23	0.54	0.97
	Phe.	0.00	2.19	0.33	0.01
	Ant.	0.44	1.07	0.12	0.18
% of 3 rings/total PAHs		0.92	17.43	13.93	8.38
4	Flu.	30.41	8.07	18.99	24.89
	Pyr.	50.30	15.68	36.75	48.08
	BaA.	4.70	6.02	13.68	17.69
	Chr.	41.61	8.71	19.89	25.92
% of 4 rings/total PAHs		44.83	46.73	48.85	47.47
5	BbF.	26.11	10.53	9.11	18.67
	BkF.	19.74	1.78	0.00	12.28
	BaP.	1.99	5.62	3.16	11.32
	DahA.	61.68	11.59	39.66	59.36
% of 5 rings/total PAHs		38.65	35.84	28.40	41.38
6	Bp.	24.57	0.00	16.09	1.81
	Indeno	19.24	0.00	0.05	0.00
% of 6 rings/total PAHs		15.46	0.00	8.82	0.74
	Total PAHs	283.36	82.36	182.83	245.62
% degradation of total PAHs			70.93	35.48	13.32

## Data Availability

The data used to support the findings of this study are included within the article.
